# The small molecule inhibitor 3PO is a modulator of neutrophil metabolism, ROS production, and NET release

**DOI:** 10.1093/cei/uxaf012

**Published:** 2025-02-19

**Authors:** Michele Fresneda Alarcon, Genna Ali Abdullah, Andy Nolan, Christina Linford, Maria Martina Meschis, Andrew L Cross, Andrew Sellin, Marie M Phelan, Helen L Wright

**Affiliations:** Institute of Life Course and Medical Sciences, University of Liverpool, Liverpool, UK; Institute of Life Course and Medical Sciences, University of Liverpool, Liverpool, UK; Institute of Life Course and Medical Sciences, University of Liverpool, Liverpool, UK; Institute of Life Course and Medical Sciences, University of Liverpool, Liverpool, UK; Institute of Systems, Molecular and Integrative Biology (ISMIB), Liverpool Head and Neck Centre, University of Liverpool, Liverpool, UK; Institute of Life Course and Medical Sciences, University of Liverpool, Liverpool, UK; Institute of Life Course and Medical Sciences, University of Liverpool, Liverpool, UK; Institute of Systems Molecular and Integrative Biology, University of Liverpool, Liverpool, UK; High Field NMR Facility, Liverpool Shared Research Facilities, University of Liverpool, Liverpool, UK; Institute of Life Course and Medical Sciences, University of Liverpool, Liverpool, UK

**Keywords:** neutrophils, metabolomics, PFKFB3, glycolysis, rheumatoid arthritis

## Abstract

Neutrophils are key effector leukocytes of the innate immune system and play a pivotal role in defending the host against microbial infections. Recent studies have identified a crucial link between glycolysis and neutrophil cellular functions. Using human neutrophils, we have investigated the intricate relationship between glycolysis, extracellular glucose availability, and the enzyme 6-phosphofructo-2-kinase/fructose-2,6-bisphosphatase 3 (PFKFB3), in the regulation of reactive oxygen species (ROS) and neutrophil extracellular trap (NET) production. We have identified that PFKFB3 is elevated in rheumatoid arthritis (RA) neutrophils and that the small molecule PFKFB3 inhibitor 3PO is a key regulator of neutrophil ROS and NET production. 3PO blocked the production of ROS and NETs in a dose-dependent manner in both RA and healthy neutrophils (*P* < 0.01), and RA neutrophils were more sensitive to lower concentrations of 3PO. Bacterial killing was only partially inhibited by 3PO, and the proportion of live neutrophils after 24 h incubation was unchanged. Using NMR metabolomics, we identified that 3PO increases the concentration of lactate, phenylalanine, and L-glutamine in neutrophils, as well as significantly decreasing intracellular glutathione (adj. *P*-value < 0.05). We also demonstrated that RA neutrophils produce ROS and NETs in culture conditions which mimic the low glucose environments encountered in RA synovial joints. Our results also suggest that 3PO may have molecular targets beyond PFKFB3. By dissecting the intricate interplay between metabolism and neutrophil effector functions, this study advances the understanding of the molecular mechanisms governing pro-inflammatory neutrophil responses and identifies 3PO as a potential therapeutic for conditions characterized by dysregulated neutrophil activation.

## Teaser

The small molecule inhibitor 3PO blocks pathogenic reactive oxygen species (ROS) and neutrophil extracellular trap (NET) production by neutrophils. Using human neutrophils, we have investigated the intricate relationship between glycolysis, extracellular glucose availability, and the enzyme 6-phosphofructo-2-kinase/fructose-2,6-bisphosphatase 3, in the regulation of ROS and NET production.

## Graphical Abstract

**Graphical Abstract:**
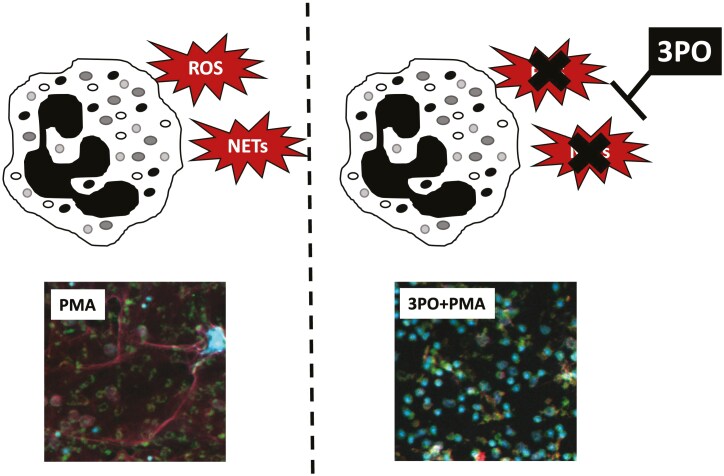

## Introduction

Complex changes in leukocyte metabolism are associated with the generation of small molecule metabolites, such as ATP, NADPH, nucleotides, and amino acids, which are required rapidly and in high abundance for cell activation, migration, and differentiation [1]. Understanding these metabolic networks and adaptations has become more relevant since the discovery of the impact of metabolism on immunity (immunometabolism) and the realization that cellular metabolism does much more than simply provide energy or biomass. Metabolite and metabolic fluxes can modulate activation or inhibition of cell signalling pathways and post-translational modifications to proteins [2].

Dysregulation of immunometabolic control has been described in inflammatory diseases including rheumatoid arthritis (RA), where changes in T-cell glycolytic activity drive differentiation, hyper-proliferation, and hyper-migration of T-cell subsets [3–5]. RA macrophages have a disease specific metabolic signature, which enables CD4^+^ T cells to differentiate into hyperproliferative pro-inflammatory helper T cells invading tissue and eliciting aggressive tissue inflammation in the synovium through immunogenic cell death [6]. The loss of self-tolerance in people with RA precedes joint inflammation by decades and is a consequence of metabolic dysregulation of both the innate and adaptive immune systems [7].

Neutrophils are the most abundant leukocyte in humans and are specialist cells of the innate immune system that play a major role in host defence against micro-organisms through phagocytosis and generation of ROS [8, 9]. Neutrophils have enormous potential to cause damage to local tissues when dysregulated and are key mediators of inflammation and cartilage damage in RA [10]. Neutrophil survival is enhanced in inflammatory conditions by hypoxia-driven metabolic re-programming [11] and by nutrient availability at the site of inflammation [12]. Neutrophils rely on glycolysis to fuel their energy requirements, where glucose is converted into pyruvate in the cytosol for relatively low-level production of ATP and NADH [13]. The resulting pyruvate is not oxidized in the mitochondria through the TCA cycle, rather it is converted to lactate enabling the generation of NAD^+^ for re-use in the glycolytic pathway. Furthermore, inflammatory neutrophils contain glycogen, stores of which can be modified when circulating neutrophils are recruited into tissues, by activation of the oxygen-sensing response, and by stimulation with proinflammatory mediators [14, 15]. The first intermediate of glycolysis, glucose 6-phosphate (G6P), also fuels the pentose phosphate pathway (PPP) producing NAPDH which is required to activate the NADPH oxidase (NOX2) leading to ROS production, chromatin decondensation, NOX2-dependent NET formation, and NET release [16, 17].

We recently measured the neutrophil metabolome in RA using ^1^H NMR metabolomics and identified lower levels of intracellular glucose and higher levels of lactate and NADP^+^ in RA neutrophils compared with healthy controls [18]. The reliance on glycolysis for cellular activation is a challenge for neutrophils as sites of inflammation, such as the RA synovial joint, are frequently characterized by limited nutrient availability, including glucose as well as hypoxia [19]. The aim of this work was to further examine the pathways of central carbon metabolism, i.e. glycolysis, gluconeogenesis, and glutaminolysis to determine the importance of these in pathogenic neutrophil release of ROS and NETs in RA.

## Materials and methods

### Isolation and incubation of neutrophils

Ultrapure neutrophils (>99.9% purity) were isolated using the untouched neutrophil isolation kit (Stem Cell). After the blood was incubated 1:5 ratio with Hetasep (Stem Cell) for 30 min at 37°C, the upper layer of nucleated cells was collected and resuspended 1:1 with ice cold isolation buffer (PBS containing 2% BSA and 0.2-mM EDTA). The suspension was then centrifuged at 400 g for 5 min and pellet resuspended in 2 ml of isolation buffer. Twenty-five microlitres of the antibody cocktail was added to the cell suspension and then incubated for 10 min on ice before adding 50 mL of magnetic beads and further incubating for 10 min in an EasySep magnet. Cells were then collected and resuspended in RPMI media (+L-glutamine, −HEPES, −phenol red) containing either glucose (11 mM) or no glucose as indicated at 5 × 10^6^/ml. In some experiments, neutrophils were pre-treated with 3PO, BPTES, MB05032, or CP-91149 at a range of concentrations (10–50 mM), 2-DG (50 mM), or AZ-PFKFB3-67 (10nM–10 mM) for 15 min prior to activation with PMA (concentrations below).

### Detection of ROS production by luminol-enhanced chemiluminescence

Following incubation, 2 × 10^5^ cells were resuspended in 200-mL HBSS along with 1-mL luminol (final concentration 10 mM). The respiratory burst was stimulated with PMA (100 ng/mL) and measured in a Fluostar plate reader luminometer at 37°C.

### Measurement of NET production with Sytox green

To determine the formation of NETs, 10^5^ neutrophils were seeded in a black 96-well plate containing Sytox green reagent (5 mM) and treated firstly with or without cell signalling inhibitors for 15 min in a humidified incubator at 37^o^C. Then, neutrophils were stimulated with PMA (600 nM) and incubated for 4 h at 37^o^C before fluorescence was measured by Fluostar plate reader at excitation 488 nm and emission in the 530-nm filter.

### Imaging of neutrophils on cover slips

Neutrophils were seeded (at 2 × 10^5^ cells/500 μL) in RPMI media in a 24-well plate containing poly-L-lysine coated coverslips, as previously described [20]. Cells were pre-treated with 3PO (50 mM) or left untreated and allowed to settle and adhere for 30 min prior to stimulation with PMA (600 nM). Cells were incubated for a further 4 h to allow for NET production. Cells adhered to coverslips were fixed with 4% paraformaldehyde prior to immunofluorescence staining. Briefly, coverslips were removed from the plate and washed with PBS, permeabilized with 0.05% Tween 20 in TBS, fixed with TBS (+2% BSA), and then stained for 30 min on drops of TBS (+2% BSA) on parafilm stretched across a clean 24-well plate. Primary antibodies used were mouse anti-myeloperoxidase (1:1000, Abcam) and rabbit anti-elastase (1:200, Abcam). Coverslips were washed three times with TBS prior to secondary antibody staining (anti-rabbit AlexaFluor488 and anti-mouse AlexaFluor647, 1:2000, Life Technologies) in TBS (+2% BSA) for 30 min. Coverslips were washed prior to staining with DAPI (1 μg/ml). Slides were imaged on a Zeiss LSM800 microscope (Zeiss) using a 20× objective.

### Measurement of apoptosis by flow cytometry

Following incubation with or without inhibitors for up to 24 h, 5 × 10^4^ neutrophils were removed from culture and diluted with 200-ml HBSS containing 0.5-ml Annexin V-488 and incubated at room temperature in the dark for 15 min. The total volume was then made up to 250 ml with HBSS and 0.25-ml propidium-iodide added (1 mg/ml, final conc) before reading on a EasyCyte Guava flow cytometer. Five thousand events were analysed per sample.

### Measurement of GLUT3 expression by flow cytometry

Neutrophils (5 × 10^4^) were labelled with saturating concentrations of FITC-labelled anti-GLUT3 antibody or isotype control (BD Biosciences). Unlabelled cells were also prepared as a control. Cells were incubated in PBS (0.2% BSA) with or without antibodies for 30 min at 4^o^C. Cells were washed in PBS (BSA) and then fixed using 4% (final conc) paraformaldehyde in PBS. Cells were washed again to remove excess paraformaldehyde and then resuspended in 200-ml PBS. Cell surface antibodies were detected using a Cytoflex Beckman Coulter 2.0 flow cytometer, with 10 000 cells counted per sample.

### NMR metabolomics

Neutrophils were prepared for metabolite extraction as previously described [18]. Briefly, cells were centrifuged at 1000 g at 25^o^C for 2 min and the pellet washed with ice cold PBS then centrifuged at 1000 g at 25^o^C for 2 min. The supernatant was discarded, while pellets were heated at 100^o^C for 1 min, and then snap-frozen in liquid nitrogen and stored at −80^o^C. Metabolites were extracted by addition of 50:50 v/v ice cold HPLC grade acetonitrile:water at 500 μl per cell pellet, followed by a 10-min incubation on ice. Samples were sonicated while incubated in an ice water bath for three 30 s bursts at 23 kHz and 10-μm amplitude using an exponential microtip probe, with 30-s rest between sonications. Sonicated samples were centrifuged at 12 000g for 5 min at 4^o^C and the supernatant transferred to cryovials, flash frozen in liquid N_2_ and lyophilized prior to storage at -80^o^C. Each lyophilized sample was resuspended in 200 μl of 100 μM deuterated sodium phosphate buffer pH 7.4, with 100 μM trimethylsilyl propionate (TSP) and 0.05% NaN_3_. Each sample was vortexed for 20 s and centrifuged at 21 500 g for 5 min at 5^o^C. Then, 180 μl of each cell extract sample was transferred to 3-mm (outer diameter) NMR tubes for acquisition. The samples were analysed using a 700-MHz NMR Avance IIIHD Bruker NMR spectrometer equipped with a TCI cryoprobe. Samples were referenced to trimethylsilylpropanoic acid (TSP) at 0 ppm. Spectra were acquired at 25^o^C using the 1D ^1^H Carr–Purcell–Meiboom–Gill (CPMG) edited pulse sequence (vendor supplied cpmgpr1d) with 512 scans and a 4-s interscan delay and 1.8-s acquisition time. Spectra were proceed using vendor supplied auto routine (apk0.noe) prior to manual acceptance via quality control criteria. Spectra were assessed to conform to minimum quality criteria as outlined by the Metabolomics Standards Initiative [21] to ensure linewidths at half height within 1 SD with consistent signal-to-noise, baseline correction, and water suppression. All spectra passing quality criteria were then divided into peak boundaries ‘bins’ that were defined globally by the peak limits using Chenomx NMR Suite 8.2 (Chenomx Inc., Edmonton, Alberta, Canada) [22]. All peaks, both annotated in Chenomx (via manual analyses in TopSpin and Chenomx software) and unknowns, were included in the bin table. A correlation-reliability scoring (CRS) method developed by Grosman [23] was applied to the data. This method takes a representative metabolite peak based on the premise that each individual metabolite exhibits an ^1^H NMR spectrum comprising multiple peaks that are highly correlated across the spectra [23]. Raw data, metabolite annotation, and NMR parameters are deposited in the MetaboLights repository (MTBLS6215) [24].

### Preparation of immune complexes

Insoluble immune complexes (IICs) were prepared by incubating human serum albumin with anti-human serum albumin antibody in PBS for 1 h as previously described [25]. Immune complexes were washed 3 times by centrifugation at 1000 g and re-suspended in PBS before use to remove soluble immune complexes. In some experiments, neutrophils were pre-treated with 3PO (0–50 mM) for 15 min prior to stimulation with 10% (v/v) IIC to stimulate NET production.

### Western blotting

Neutrophils were boiled in Laemmli buffer containing 50-mM DTT at 95°C for 5 min.

Proteins were separated by SDS-PAGE using a NuPAGE bis-Tris 4–12% gradient gel and transferred into a PDVF membrane using the Trans-Blot Turbo apparatus (Bio-Rad). Membranes were blocked in 5% BSA in Tris-buffered saline-Tween 20 (TBS-T), prior to overnight incubation at 4°C with primary antibodies: PFKFB3 (1:1000, Abcam) and Actin (1:10 000, Abcam). Blots were then washed in TBS-T prior to incubation with anti-rabbit IgG HRP secondary antibody (1:10 000, Cell Signaling) and anti-mouse IgG HRP secondary antibody (1:10 000, Sigma). Bands were detected using enhanced chemiluminescent substrate (ECL; Thermo Fisher, Loughborough, UK) and imaged on a ChemiDoc image analyser (Bio-Rad Laboratories). Densitometry analysis was performed using Fiji [26].

### Bacterial killing assay

Bacterial killing assay was performed as previously described [27]. Briefly, freshly grown *Staphylococcus aureus* were harvested and washed, and suspended at 5 × 10^8^/ml in HBSS and opsonized with 10% human AB serum (Sigma) for 30 min at 37°C. Freshly isolated neutrophils (10^6^/ml) were primed with TNFa (10 ng/ml) and incubated for 1 h at 37°C with gentle agitation with serum-opsonized bacteria at a ratio of 1:10. Neutrophils were then lysed to release live bacteria by serial dilution in distilled water and vigorous vortexing, before being plated onto LB agar plates and incubated overnight. Colonies were counted and results calculated as percentage of bacteria killed compared with bacteria only (no neutrophils) samples.

### Gene expression analysis

Expression of genes relating to glycolysis was measured in NCBI Gene Expression Omnibus (GEO) datasets GSE274598 and GSE274996 [28, 29]. Datasets were analysed by DESeq2 [30] to detect genes significantly different between RA (*n* = 65) and HC (*n* = 11) (adj. *P*-value < 0.05).

### Data analysis

Statistical analyses were performed using R v4.0.2. For experimental data, the Shapiro–Wilk test was used to determine normality. Then, univariate analysis was carried out by the Student’s *t*-test, Wilcox test, or ANOVA as appropriate. Multivariate ^1^H NMR metabolomics data were assessed for normality via qqplots and found to be non-normally distributed; therefore, a Wilcox test was performed with application of a False-Discovery Rate (FDR) and adjusted *P*-value of 0.05. Fold change comparisons were performed comparing natural log of median metabolite abundances on probabilistic quotient normalized data. Metabolic pathway enrichment analysis was performed using fold change analysis to select 15 metabolites with mean fold change greater than 20%. Over representation analysis was performed using Metaboanalyst v6.0 [31] against the small molecule pathway database of 99 metabolite sets based on normal human metabolic pathways. Analysis was filtered for metabolite sets containing at least 2 entries (reference metabolome based on all compounds in library).

## Results

### Increased expression of genes regulating central carbon metabolism in RA neutrophils

**Figure 1:**
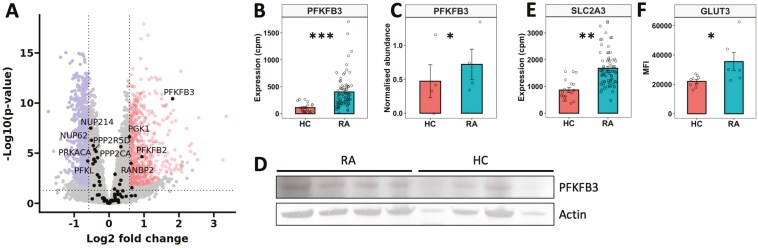
dysregulation of metabolic gene expression in RA neutrophils. (A) Volcano plot comparing gene expression in RA vs HC neutrophils, with glycolysis genes highlighted by name. Significantly higher gene expression in RA (highlighted upper right), lower expression (highlighted upper left) (FDR < 0.05). (B) Expression of PFKFB3 mRNA in RA (*n* = 65) and HC (*n* = 11) neutrophils. (C) Densitometry analysis of PFKFB3 protein by (D) western blot, normalized to actin (RA *n* = 4, HC *n* = 4). (E) Expression of SLC2A3 mRNA in RA (*n* = 65) and HC (*n* = 11) neutrophils. (F) GLUT3 protein measured by flow cytometry (MFI) on RA (*n* = 6) and HC (*n* = 9) neutrophils. (Experimental data analysed by Student’s *t*-test, *P < 0.05. RNAseq data analysed using DESeq2 **FDR < 0.05, ***FDR < 0.01)

In our previous study, we identified that the metabolome in RA neutrophils is different from HC, mainly driven by metabolites of central carbon metabolism (glucose, ATP, NADP, lactate) [18]. In this study, we wanted to further investigate these differences and how altered metabolism might contribute to pathogenic neutrophil functions. We first investigated the expression of genes from the glycolysis pathway in our recently published gene expression datasets (NCBI GEO GSE274598, GSE274996) [29]. We found that expression levels of several genes relating to glycolysis were significantly higher in RA neutrophils (Fig. 1A). The largest difference between RA and HC neutrophils was the expression of the gene for 6-Phosphofructo-2-Kinase/Fructose-2,6-Bisphosphatase 3 (PFKFB3) which was significantly higher in RA neutrophils (FDR = 0.01, Fig. 1B). We confirmed increased protein abundance of PFKFB3 in RA neutrophils by western blotting (Fig. 1C, D, *P* < 0.05). We also detected increased gene expression levels of SLC2A3 (Fig. 1E FDR = 0.04). This encodes the glucose transporter GLUT3, and increased abundance of GLUT3 protein on RA neutrophils was confirmed by flow cytometry (Fig. 1F, *P* < 0.05).

### Absence of extracellular glucose does not change ROS or NETs in RA neutrophils, but inhibition of glycolysis blocks ROS and NET production in both HC and RA neutrophils

**Figure 2:**
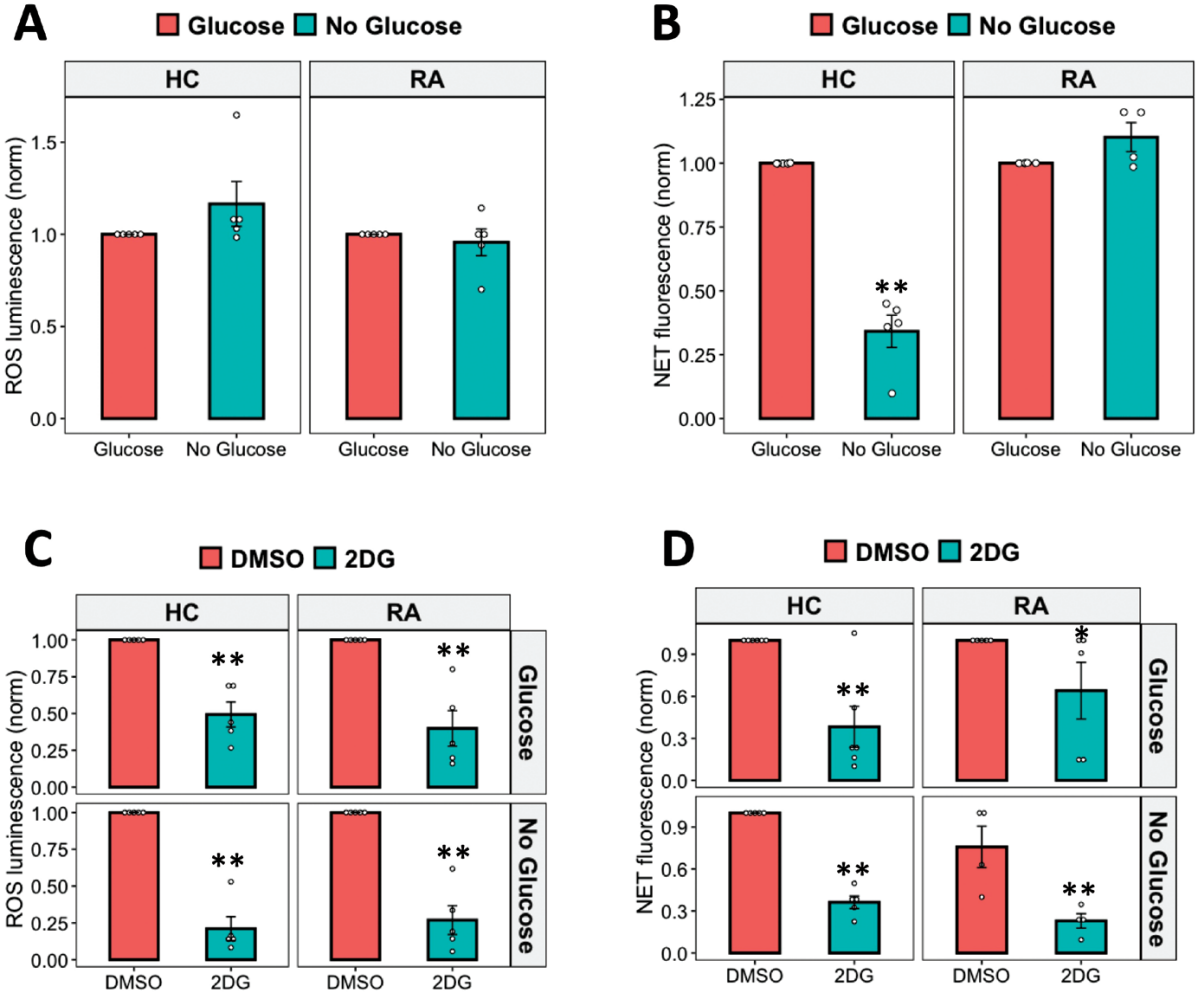
effect of glucose availability on ROS and NET production. (A) ROS and (B) NET production in response to PMA were measured in glucose-containing or no glucose media. The effect of hexokinase inhibition with 2-DG on neutrophil (C) ROS and (D) NET production was measured in glucose-containing or no glucose media (Data analysed by Student’s *t*-test. *P < 0.05, **P < 0.01, *n* = 4–6)

Neutrophils are often found at high concentrations in inflamed sites, e.g. in RA synovial joints where glucose concentrations are much lower than in blood [19]. In order to investigate the requirement for extracellular glycolysis to fuel ROS and NET production, we incubated HC and RA neutrophils in media containing 11-mM glucose (glucose-containing) to model the blood environment or media without glucose (no glucose) to model synovial joints. Neutrophils were activated with PMA to stimulate NET and ROS production. The absence of glucose in the media did not affect neutrophil ROS production (Fig. 2A) but did decrease NET production by HC neutrophils (Fig. 2B, *P* < 0.01). In contrast, RA neutrophil NETs were not affected by the absence of extracellular glucose and were significantly higher than HC NETs in no glucose media (Supplementary Fig. 1, *P* < 0.01), indicating an adaptation to preserve NET production in low-glucose environments. Competitive inhibition of the first step of glycolysis (the production of glucose-6-phosphate by hexokinase) using 2-deoxy-D-glucose (2-DG) significantly decreased ROS production by both HC and in RA neutrophils (Fig. 2C, *P*-value < 0.01). 2-DG also completely inhibited NET production in glucose-containing or no glucose media in both HC and RA neutrophils (Fig. 2D, *P*-value < 0.01).

### The chemical inhibitor 3PO blocks ROS and NET production by RA and HC neutrophils

**Figure 3:**
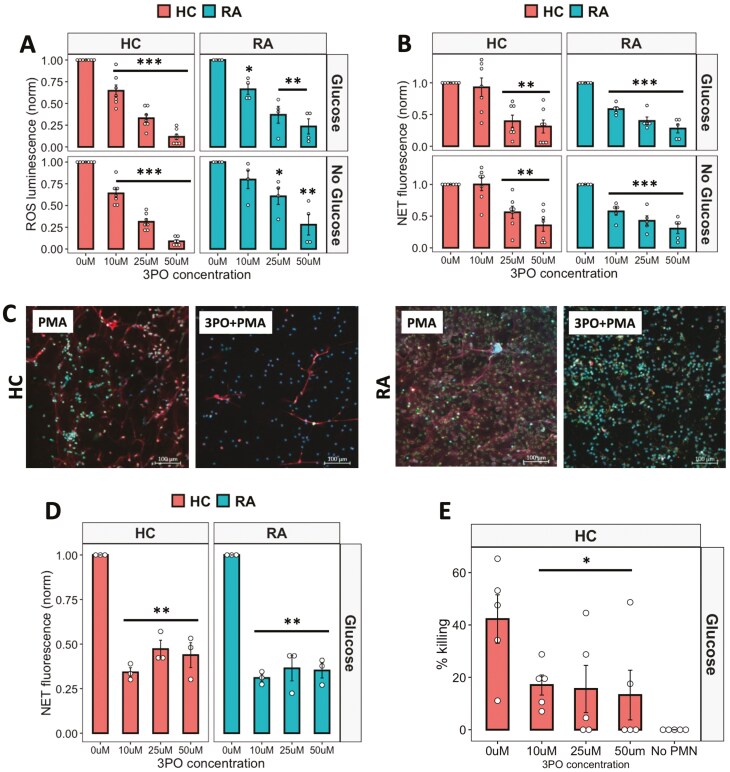
anti-inflammatory effect of 3PO on neutrophils. (A) ROS (B) and NET production in response to PMA by neutrophils incubated with 3PO (0–50 mM) in glucose-containing or no glucose media (RA *n* = 5, HC *n* = 7, *P < 0.05, **P < 0.01, ***P < 0.001). (C) Reprsentative images of HC and RA NETs produced in response to PMA in the absence or presence of 3PO (50mM). Blue = DNA, DAPI, Red = MPO, Green = elastase. Scale bars indicate 100 mM. (D) NET production in response to IICs incubated with 3PO (RA *n* = 3, HC *n* = 3, 0–50 mM, **P < 0.01). (E) Killing of *S. aureus* by 3PO-treated neutrophils (HC *n* = 5, 0–50 mM, *P < 0.05) compared with no neutrophil (No PMN) control. Data analysed by one-way ANOVA with Tukey’s post-hoc test

**Figure 4:**
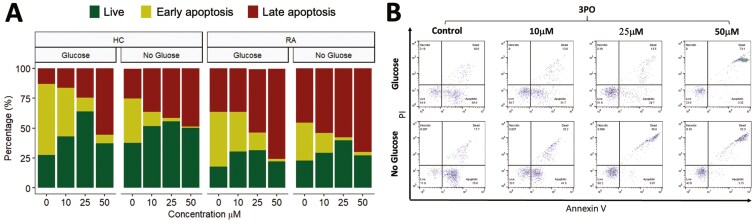
effect 3PO on neutrophil apoptosis. (A) Percentage of late apoptosis (red), early apoptosis (yellow), or live (green) HC and RA neutrophils (*n* = 3 each) incubated in glucose or no glucose media and treated for 24 h with varying concentrations of 3PO (0–50 mM). (B) Representative flow cytometry traces for HC neutrophils showing annexin V and PI positive/negative gating

Fructose-2,6-phosphate (PFK-2) is a key enzyme in the early stages of glycolysis, and our gene expression analysis identified that the PFK-2 subunit PFKFB3 was significantly increased in RA neutrophils (Fig. 1B). We therefore inhibited PFK-2 activity using 3PO, a chemical inhibitor of the PFKFB3 subunit. ROS production was inhibited in a dose-dependent manner (0–50 mM) in both HC and RA neutrophils (Fig. 3A, *P* < 0.01). In HC and RA neutrophils, increasing concentrations of 3PO significantly decreased ROS production in both types of media. RA neutrophil NET production was also significantly inhibited at all concentrations from 10 to 50 mM (*P* < 0.001). However, in HC neutrophils, 3PO did not inhibit NETs at a concentration of 10 mM, and a significant decrease was only observed at the higher concentrations of 25 and 50 mM (Fig. 3B,C, *P* < 0.01) indicating RA neutrophils may be more sensitive to 3PO treatment. 3PO inhibition of NETs was confirmed by immunofluorescence staining of fixed neutrophils on coverslips after 4-h incubation in glucose-containing media with PMA in the absence or presence of 3PO (50 mM) (Fig. 4C). We also determined that 3PO significantly inhibited NET production at all concentrations in RA and HC neutrophils in response to immune complexes (IIC) which mimic auto-antibody immune complexes such as rheumatoid factor, commonly found in the serum and synovial fluid of people with RA (Fig. 3D, *P* < 0.01). Finally when neutrophils were pre-treated with 3PO (0–50 mM), we observed a dose-dependent decrease, but not complete inhibition, of bacterial killing capacity (Fig. 3E, *P* < 0.05).

Next, we determined the effect of 3PO on neutrophil apoptosis by incubating RA and HC neutrophils for 24 h in the absence and presence of 3PO. Interestingly, in both glucose-containing and no glucose media, we observed no difference in the overall percentage of live (annexin V negative, PI negative) neutrophils. However, the populations of early apoptotic (annexin V positive, PI negative) and late apoptotic (annexin V positive, PI positive) cells were significantly decreased and increased respectively in both RA and HC by 3PO (Fig. 4A, *P* < 0.05).

### 3PO may exert an off-target anti-inflammatory effect in human neutrophils

**Figure 5:**
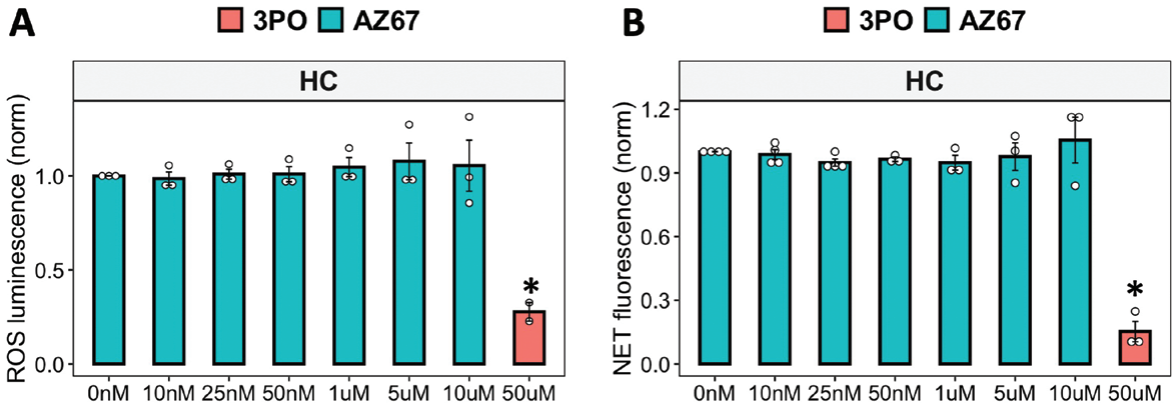
inhibition of PFKFB3 in neutrophils using AZ67. (A) ROS and (B) NET production in response to PMA by neutrophils incubated with AZ-PFKFB3-67 (0–10 mM) in glucose media, with 3PO (50 mM) as positive control (HC *n* = 3–4, *P < 0.05). Data analysed by one-way ANOVA with Tukey’s post-hoc test

Recent reports on the use of 3PO to inhibit PFKFB3 have shed doubt on the specificity of this compound to bind to its reported target [32, 33]. Indeed, recent publications have suggested that whilst 3PO does inhibit glycolysis, this is not in response to PFKFB3 binding and inhibition [32, 33]. Off-target inhibition of NF-kB by 3PO has also been reported [33]. We therefore decided to repeat the ROS and NET assays using an alternative inhibitor of PFKFB3, AZ-PFKFB3-67 (AZ67). This compound is reported to be a highly specific inhibitor of PFKFB3 with an IC_50_ of 11nM. We found that pre-treatment of HC neutrophils with increasing concentrations of AZ67 (10 nM–10 mM) had no effect on neutrophil ROS or NET production (Fig. 5A, B), in contrast to our results with 3PO.

**Figure 6:**
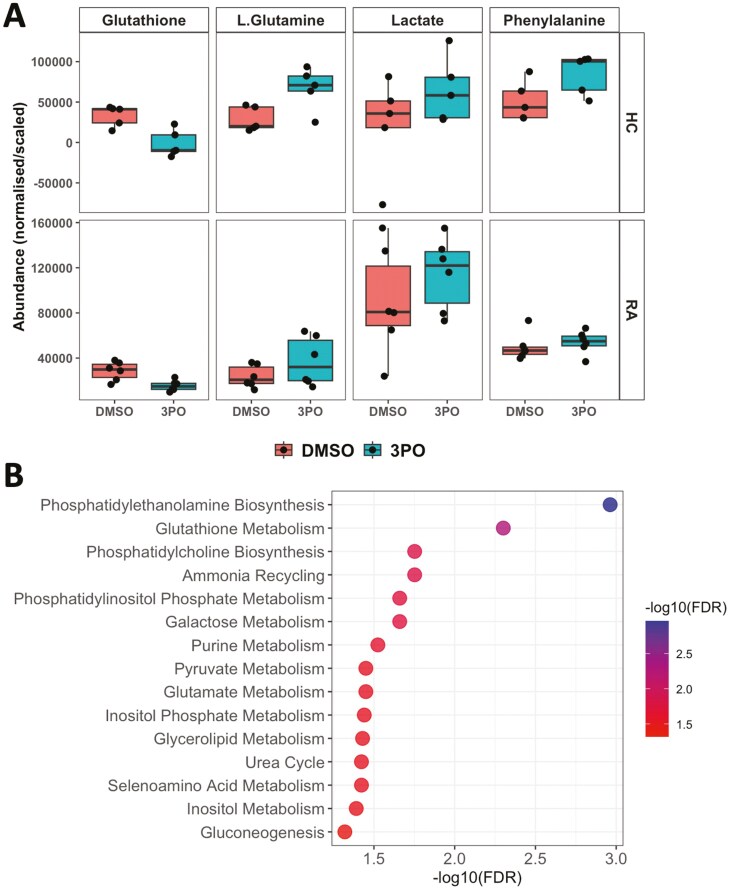
^1^H NMR metabolomics analysis of RA and HC neutrophils with PFKFB3 inhibition by 3PO. (A) Boxplots of metabolites measured by ^1^H-NMR from RA (*n* = 6) and HC (*n* = 5) neutrophils incubated in glucose containing media for 4 h with and without treatment with 50μM 3PO. (B) Pathway enrichment analysis results showing pathways with at least 3 metabolites and FDR-adjusted *P*-value < 0.05. Data analysed by Wilcox test with Benjamini–Hochberg *P*-value adjustment

In order to determine the molecular mechanism of ROS and NET inhibition by 3PO, we performed ^1^H NMR metabolomics analysis on neutrophils incubated for 4 h in the absence or presence of 3PO (50 mM) in glucose-containing media. We found that 3PO had a more profound effect on HC neutrophils than on RA neutrophils. The levels of glutathione were significantly decreased in RA and HC neutrophils (Fig. 6A, adj.*P* < 0.05). Mean L-glutamine levels increased by 2.3-fold in HC and 1.6-fold in RA neutrophils, whereas mean phenylalanine levels increased by 1.7-fold in HC neutrophils but only 1.1-fold in RA neutrophils (Fig. 6A, adj.*P* > 0.05). Mean lactate levels increased by 3.0-fold in HC neutrophils and 1.3-fold in RA neutrophils after 4-h 3PO treatment (Fig. 6A, adj.*P* > 0.05). Pathway enrichment analysis of metabolites that increased or decreased by at least 20% in response to 3PO was performed using MetaboAnalyst and is summarized in Fig. 6B. Pathways that were significantly over represented in metabolite variance associated with 3PO included those relating to membrane lipid synthesis, glutathione metabolism, and amino acid recycling.

### Inhibitors of gluconeogenesis and glutaminolysis do not affect ROS and NET production in RA and HC neutrophils

**Figure 7:**
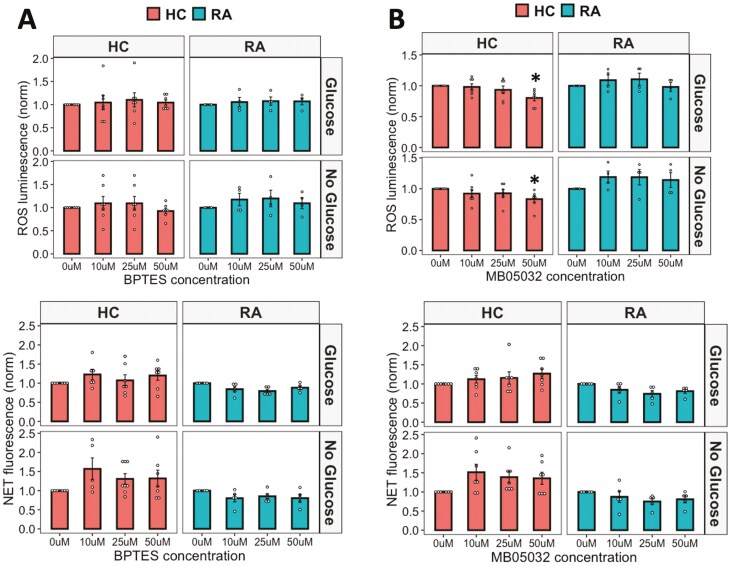
effect of glutaminolysis and gluconeogenesis on neutrophil activation. (A) ROS and (B) NET production in response to PMA by RA and HC neutrophils in glucose-containing or no glucose media when treated with an inhibitor of gluconeogenesis MB05032 or an inhibitor of glutaminolysis BPTES (0–50 mM) (RA *n* = 5, HC *n* = 8, *P < 0.05). Data analysed by ANOVA with Tukey’s post-hoc test

Finally, we investigated the effect of inhibiting gluconeogenesis and glutaminolysis on ROS and NET production by RA and HC neutrophils. MB05032 is a potent FBPase (FBP1) inhibitor which inhibits gluconeogenesis and BPTES inhibits glutaminase-1. MB05032 inhibited HC NETs at the highest concentration tested (50 mM, *P* < 0.05), but otherwise neither inhibitor significantly inhibited ROS or NET production in RA or HC neutrophils in glucose-containing or no glucose media (Fig. 7).

## Discussion

Recent studies have highlighted the importance of pathways connected to glycolysis in neutrophils, including glycogenolysis, gluconeogenesis, glutaminolysis, and PPP [17, 18]. Recent analysis of RA neutrophils by ^1^H NMR metabolomics identified significantly elevated levels of energy metabolites (ATP, ADP, GTP, NADP) and metabolites of redox homeostasis (glutathione, taurine) in RA neutrophils [18]. Lower levels of intracellular glucose in RA neutrophils were accompanied by higher levels of lactate suggesting altered glycolytic activity associated with inflammatory disease [18]. The work presented in this manuscript has used a combination of cell signalling inhibitors and media with or without glucose to mimic the inflammatory environment and provide new information about the critical role of metabolism in regulating ROS production, NET release, and apoptosis in human neutrophils.

Neutrophils are the first responders to inflamed sites. These environments generally have lower glucose concentrations than peripheral blood, a potentially unfavourable environment for neutrophils given their reliance on glycolysis for energy production and activation [16]. However, neutrophils are often found at high concentrations in low glucose, inflamed sites, e.g. in synovial fluid from people with RA [19]. Our first experiments explored neutrophil ROS and NET production in a model of normal blood and low-glucose environments. We confirmed that neutrophils do require glycolysis for ROS and NET production, given the complete inhibition of both in HC and RA neutrophils by 2-DG as previously reported. However, the absence of glucose in the extracellular media did not inhibit ROS production. NET formation is dependent on ROS production and the inhibition of intragranular MPO activity has been shown to correlate with the inhibition of NET formation [34–36]. When measuring NET formation at a single timepoint of 4 h, no differences were observed between glucose-containing and no glucose media. However, complete inhibition of glycolysis by 2-DG prevented NET formation. This could highlight that intracellular ROS formation, irrespective of the extracellular glucose environment, is sufficient for processing by MPO in order to trigger NET formation.

Analysis of our published transcriptomics data from RA and HC neutrophils provided an extra layer of insight into the dysregulation of metabolic pathways in RA neutrophils [29]. We identified increased expression of several glycolytic genes in RA neutrophils, the most significant of which was PFKFB3, an isoform of the protein PFK-2. Four different PFK-2 isozymes have been identified. Their expression is tissue-specific, and the overexpression of two isozymes (PFKFB3 and PFKFB4) has been demonstrated in various solid tumours and haematological cancer cells [37]. Peripheral and tissue resident T-cells from people with RA have a unique metabolic signature with an impairment of glycolysis due to a deficiency of PFKFB3, resulting in delayed glycolysis and increased PPP via the up-regulation of glucose-6-phosphate dehydrogenase (G6PD) [3]. The ratio of these two enzymes in T cells correlates to disease activity in people with RA, which suggests a dysregulation of pro-inflammatory properties of T cells directly affected by PFKFB3 and G6PD [4, 38]. T cells are therefore diverting glucose from energy generation toward synthesis of biomass precursors with functional consequences that include hyper-proliferation, G2/M bypass, and deviated functional commitment [39].

Little is known about the role of PFKFB3 in regulating neutrophil metabolism and function. Enhancement of PFKFB3 transcription and translation facilitates the production of neutrophil inflammatory factors during the acute phase of sepsis [40]. Pharmacological inhibition or depletion of PFK-2 by small interfering RNA (siRNA) leads to a significant decrease in NOX2 activity in neutrophils in response to various stimuli [41]. Among the four isoenzymes of PFK-2, PFKFB3 and PFKFB4 are the two main isoenzymes expressed in human neutrophils. PFKFB3 is a critical glycolysis regulatory enzyme that promotes fructose 2,6-bisphosphate (F2,6BP) production which in turn activates PFK-1, increasing glycolytic output. It has the highest kinase:phosphatase activity ratio of all the PFKFB isoforms [42], proving a possible explanation as to why inhibition of this isoform has such a marked effect on cellular function. *In vitro* experiments showed that glycolytic metabolism with PFKFB3 involvement supports inflammatory cytokine expression [40]. Furthermore, a significant increase has been observed in the expression of PFKFB3 in LPS-challenged and sepsis neutrophils compared with healthy controls [43].

Little else is known about the role of phosphofructokinase enzymes in neutrophils. For this reason, we tested whether neutrophils were still able to activate the NOX2 pathway to produce ROS and NETs when challenged with the PFKFB3 inhibitor 3PO. We observed a concentration-dependent inhibition of ROS production by 3PO in HC and RA neutrophils in both glucose-containing and no glucose media. NET production was also inhibited by 3PO, although RA neutrophils were more sensitive to 3PO inhibition at lower concentrations than HC neutrophils. Importantly, 3PO inhibited NET production in response to immune-complexes (IIC) which mimic the auto-immune antibodies found in the majority of people with RA. Measurement of neutrophil apoptosis after 24 h showed that increasing concentrations of 3PO increased the number of late apoptotic cells and decreased the early apoptotic cell count. The increase in late apoptotic cells at the highest concentrations of 3PO may be undesirable as these cells have leaky membranes and are likely to be pro-inflammatory especially at inflammatory sites. We also observed that bacterial killing was suppressed by 3PO. However, bacterial killing was not completely abrogated even at relatively high 3PO concentrations, indicating that the neutrophils retained some phagocytosis and killing functions despite ROS and NETs having been inhibited.

Recent doubt has been cast on the specificity of 3PO for its reported target PFKFB3 [32, 33]. When we repeated our experiments with an alternative PFKFB3 inhibitor (AZ67), we observed no effect on ROS or NET production, leading us to consider that 3PO may have an off-target, anti-inflammatory effect in human neutrophils. Our NMR metabolomics analysis confirmed an increase in lactate in response to 3PO, which aligns with recent reports [32]. We also observed a significant decrease in glutathione, which is an important metabolite in redox homeostasis. It is essential for defending cells against oxidative damage, including from ROS such as hydrogen peroxide and superoxide produced during the neutrophil respiratory burst. It acts directly as an antioxidant and as a substrate for glutathione peroxidases, which break down peroxides and prevent oxidative damage to biomolecules [44]. The decrease in glutathione observed in our experiments is likely in direct response to the decrease in ROS production in the presence of 3PO. The increase in mitochondrial metabolites L-glutamine and phenylalanine, along with the predicted increase in amino acid-recycling, may represent a metabolic switch adopted by neutrophils to compensate for the inhibition of glycolysis. L-glutamine is a significant energy source for cells of the immune system, where it is converted to glutamate and then into alpha-ketoglutarate, before entering the TCA cycle to produce ATP [45]. In rat neutrophils, glutamine enhances ROS production and regulates NADPH oxidase activity [46]. It is interesting therefore that the levels of glutamine increase in response to 3PO, and yet, ROS production is completely inhibited. Phenylalanine and its metabolite tyrosine are degraded to fumarate and acetoacetate, which are also intermediates in the TCA cycle, thus contributing to the production of ATP in the absence of glycolysis. Whilst we did not detect TCA cycle activity in PMA-activated neutrophils in our Seahorse experiments, it may be that some TCA enzymes are functional in neutrophils and may become activated in conditions of glycolysis inhibition. Further experiments should confirm whether intermediary enzymes of the TCA cycle can become active under stress conditions in neutrophils. Metabolic pathway enrichment analysis also predicted changes in a number of metabolic pathways involving membrane phospholipids, possibly reflecting the changes in the proportion of early/late apoptotic neutrophils observed in our study.

One of the limitations of our study was that the participants with RA were receiving one or more disease-modifying anti-rheumatic drugs (DMARDs, e.g. methotrexate and hydroxychloroquine), and this may account for some of the heterogeneity in the results for the RA group. The treatment groups and exclusion criteria for HC and RA samples also lead to modest sample size that limits the effectiveness of univariate analysis methods for metabolomics datasets. Data filtering using CRS to limit analysis to annotated metabolites within the data (53 metabolites) did not wholly alleviate this issue, and therefore, it is possible that more metabolite changes could be discerned with a larger sample size. Several DMARDs have known effects on neutrophil functions, including decreasing ROS production and degranulation [9]. We recorded wider experimental variation in the RA samples, which could be explained by the effect of DMARDs, differences in donor disease activity (DAS28), or other clinical factors such as inflammation (e.g. CRP). Another significant limitation of our study is that we were also not able to conclusively determine the molecular target of 3PO in human neutrophils, and the molecular target of 3PO remains unidentified despite the efforts others [32, 33]. We were unfortunately not able to perform additional experiments with an alternative PFKFB3 inhibitor (AZ67) as part of this study. This inhibitor reportedly has high specificity for PFKFB3 inhibition in cell-free enzyme activity assays and has been shown to prevent the activation of glycolysis in mouse neurons [47]. However, AZ67 has also been shown to have off-target effects on angiogenesis that are not directly related to glycolysis inhibition [48].

In summary, we have shown in this study that neutrophils are metabolically adapted to maintain ROS production in low-glucose environments such as sites of inflammation, and that NET production is preserved in RA neutrophils in low-glucose environments. We also identified the inhibitor compound 3PO as a critical regulator of pathogenic and tissue damaging inflammatory responses by human neutrophils. Further experiments will be required to fully determine whether this is via direct inhibition of PFKFB3, or via an off-target effect, which may divert energy production away from glycolysis and toward mitochondrial metabolism. Whilst 3PO partially inhibited cytotoxic bacterial killing by neutrophils we propose that, at lower and optimized concentrations, 3PO could be considered a potential therapeutic for the treatment of immune-mediated inflammatory diseases such as RA with a strong neutrophil-driven pathology.

## Supplementary Material

uxaf012_suppl_Supplementary_Materials

## Data Availability

The raw sequencing data reported in this manuscript have been deposited in the NCBI Gene Expression Omnibus (GEO) and are accessible through GEO Series accession number GSE274598 and GSE274996. Raw NMR data, metabolite annotation, and NMR parameters are deposited in the MetaboLights repository (MTBLS6215). All other data presented in this manuscript are available upon reasonable request to the corresponding author.

## References

[1] Gaber T, Strehl C, Buttgereit F. Metabolic regulation of inflammation. Nat Rev Rheumatol 2017, 13, 267–79. doi: https://doi.org/10.1038/nrrheum.2017.37 28331208

[2] Voss K, Hong HS, Bader JE, Sugiura A, Lyssiotis CA, Rathmell JC. A guide to interrogating immunometabolism. Nat Rev Immunol 2021, 21, 637–52. doi: https://doi.org/10.1038/s41577-021-00529-8 33859379 PMC8478710

[3] Yang Z, Fujii H, Mohan SV, Goronzy JJ, Weyand CM. Phosphofructokinase deficiency impairs ATP generation, autophagy, and redox balance in rheumatoid arthritis T cells. J Exp Med 2013, 210, 2119–34. doi: https://doi.org/10.1084/jem.20130252 24043759 PMC3782046

[4] Yang Z, Shen Y, Oishi H, Matteson EL, Tian L, Goronzy JJ, et al Restoring oxidant signaling suppresses proarthritogenic T cell effector functions in rheumatoid arthritis. Sci Transl Med 2016, 8, 331ra38. doi: https://doi.org/10.1126/scitranslmed.aad7151 PMC5074090 27009267

[5] Weyand CM, Goronzy JJ. Immunometabolism in early and late stages of rheumatoid arthritis. Nat Rev Rheumatol 2017, 13, 291–301. doi: https://doi.org/10.1038/nrrheum.2017.49 28360422 PMC6820517

[6] Weyand CM, Goronzy JJ. Immunometabolism in the development of rheumatoid arthritis. Immunol Rev 2020, 294, 177–87. doi: https://doi.org/10.1111/imr.12838 31984519 PMC7047523

[7] Li Y, Goronzy JJ, Weyand CM. DNA damage, metabolism and aging in pro-inflammatory T cells. Exp Gerontol 2018, 105, 118–27. doi: https://doi.org/10.1016/j.exger.2017.10.027 29101015 PMC5871568

[8] Glennon-Alty L, Hackett AP, Chapman EA, Wright HL. Neutrophils and redox stress in the pathogenesis of autoimmune disease. Free Rad Bio Med 2018, 125, 25–35. doi: https://doi.org/10.1016/j.freeradbiomed.2018.03.049 29605448

[9] Wright HL, Moots RJ, Bucknall RC, Edwards SW. Neutrophil function in inflammation and inflammatory diseases. Rheumatol 2010, 49, 1618–31. doi: https://doi.org/10.1093/rheumatology/keq045 20338884

[10] Wright HL, Moots RJ, Edwards SW. The multifactorial role of neutrophils in rheumatoid arthritis. Nat Rev Rheumatol 2014, 10, 593–601. doi: https://doi.org/10.1038/nrrheum.2014.80 24914698

[11] Walmsley SR, Print C, Farahi N, Peyssonnaux C, Johnson RS, Cramer T, et al Hypoxia-induced neutrophil survival is mediated by {HIF}-1alpha-dependent {NF}-kappaB activity. J Exp Med 2005, 201, 105–15. doi: https://doi.org/10.1084/jem.20040624 15630139 PMC2212759

[12] Sadiku P, Willson JA, Ryan EM, Sammut D, Coelho P, Watts ER, et al Neutrophils fuel effective immune responses through gluconeogenesis and glycogenesis. Cell Metab 2021, 33, 411–23.e4. doi: https://doi.org/10.1016/j.cmet.2020.11.016 33306983 PMC7863914

[13] Sbarra AJ, Karnovky ML. The biochemical basis of phagocytosis. I. Metabolic changes during the ingestion of particles by polymorphonuclear leukocytes. J Biol Chem 1959, 234, 1355–62. 13654378

[14] Sadiku P, Willson JA, Dickinson RS, Murphy F, Harris AJ, Lewis A, et al Prolyl hydroxylase 2 inactivation enhances glycogen storage and promotes excessive neutrophilic responses. J Clin Invest 2017, 127, 3407–20. doi: https://doi.org/10.1172/JCI90848 28805660 PMC5669581

[15] Robinson JM, Karnovsky ML, Karnovsky MJ. Glycogen accumulation in polymorphonuclear leukocytes, and other intracellular alterations that occur during inflammation. J Cell Biol 1982, 95, 933–42. doi: https://doi.org/10.1083/jcb.95.3.933 7153252 PMC2112917

[16] Azevedo EP, Rochael NC, Guimarães-Costa AB, de Souza-Vieira TS, Ganilho J, Saraiva EM, et al A Metabolic shift toward pentose phosphate pathway is necessary for Amyloid Fibril- and Phorbol 12-Myristate 13-acetate-induced neutrophil extracellular trap (NET) formation. J Biol Chem 2015, 290, 22174–83. doi: https://doi.org/10.1074/jbc.M115.640094 26198639 PMC4571968

[17] Chokesuwattanaskul S, Phelan MM, Edwards SW, Wright HL. A robust intracellular metabolite extraction protocol for human neutrophil metabolic profiling. PLoS One 2018, 13, e0209270. doi: https://doi.org/10.1371/journal.pone.0209270 30571714 PMC6301625

[18] Chokesuwattanaskul S, Alarcon MF, Mangalakumaran S, Grosman R, Cross AL, Chapman EA, et al Metabolic profiling of rheumatoid arthritis neutrophils reveals altered energy metabolism that is not affected by JAK inhibition. Metabol 2022, 12, 650. doi: https://doi.org/10.3390/metabo12070650 PMC9321732 35888774

[19] Anderson JR, Chokesuwattanaskul S, Phelan MM, Welting TJM, Lian L-Y, Peffers MJ, et al 1H NMR metabolomics identifies underlying inflammatory pathology in osteoarthritis and rheumatoid arthritis synovial joints. J Proteome Res 2018, 17, 3780–90. doi: https://doi.org/10.1021/acs.jproteome.8b00455 30229649 PMC6220363

[20] Chapman EA, Lyon M, Simpson D, Mason D, Beynon RJ, Moots RJ, et al Caught in a trap? Proteomic analysis of neutrophil extracellular traps in rheumatoid arthritis and systemic lupus erythematosus. Front Immunol 2019, 10, 423. doi: https://doi.org/10.3389/fimmu.2019.00423 30915077 PMC6421309

[21] Sumner LW, Amberg A, Barrett D, Beale MH, Beger R, Daykin CA, et al Proposed minimum reporting standards for chemical analysis. Metabolomics 2007, 3, 211–21. doi: https://doi.org/10.1007/s11306-007-0082-2 24039616 PMC3772505

[22] Weljie AM, Newton J, Mercier P, Carlson E, Slupsky CM. Targeted profiling: quantitative analysis of 1H {NMR} metabolomics data. Anal Chem 2006, 78, 4430–42. doi: https://doi.org/10.1021/ac060209g 16808451

[23] Grosman R. NMR metabolic profiling of mosquito species to understand insecticide resistance. 2020. University of Liverpool. doi: https://doi.org/10.17638/03067218

[24] Haug K, Cochrane K, Nainala VC, Williams M, Chang J, Jayaseelan KV, et al MetaboLights: a resource evolving in response to the needs of its scientific community. Nucleic Acids Res 2020, 48, D440–D4. doi: https://doi.org/10.1093/nar/gkz1019 31691833 PMC7145518

[25] Fossati G, Bucknall RC, Edwards SW. Insoluble and soluble immune complexes activate neutrophils by distinct activation mechanisms: changes in functional responses induced by priming with cytokines. Ann Rheum Dis 2002, 61, 13–9. doi: https://doi.org/10.1136/ard.61.1.13 11779751 PMC1753889

[26] Schindelin J, Arganda-Carreras I, Frise E, Kaynig V, Longair M, Pietzsch T, et al Fiji: an open-source platform for biological-image analysis. Nat Methods 2012, 9, 676–82. doi: https://doi.org/10.1038/nmeth.2019 22743772 PMC3855844

[27] Cross AL, Hawkes J, Wright HL, Moots RJ, Edwards SW. APPA (apocynin and paeonol) modulates pathological aspects of human neutrophil function, without supressing antimicrobial ability, and inhibits TNFalpha expression and signalling. Inflammopharmacol 2020, 28, 1223–35. doi: https://doi.org/10.1007/s10787-020-00715-5 PMC7525285 32383062

[28] Edgar R, Domrachev M, Lash AE. Gene expression omnibus: NCBI gene expression and hybridization array data repository. Nucleic Acids Res 2002, 30, 207–10. doi: https://doi.org/10.1093/nar/30.1.207 11752295 PMC99122

[29] Fresneda Alarcon M, Abdullah GA, Beggs JA, Kynoch I, Sellin A, Cross AL, et al Complexity of the neutrophil transcriptome in early and severe rheumatoid arthritis. A role for microRNAs? MedRxiv 2024. doi: https://doi.org/10.1101/2024.12.12.24318900 PMC12210129 40590363

[30] Love MI, Huber W, Anders S. Moderated estimation of fold change and dispersion for RNA-seq data with DESeq2. Genome Biol 2014, 15, 550. doi: https://doi.org/10.1186/s13059-014-0550-8 25516281 PMC4302049

[31] Pang Z, Lu Y, Zhou G, Hui F, Xu L, Viau C, et al MetaboAnalyst 6.0: towards a unified platform for metabolomics data processing, analysis and interpretation. Nucleic Acids Res 2024, 52, W398–406. doi: https://doi.org/10.1093/nar/gkae253 38587201 PMC11223798

[32] Emini Veseli B, Perrotta P, Van Wielendaele P, Lambeir AM, Abdali A, Bellosta S, et al Small molecule 3PO inhibits glycolysis but does not bind to 6-phosphofructo-2-kinase/fructose-2,6-bisphosphatase-3 (PFKFB3). FEBS Lett 2020, 594, 3067–75. doi: https://doi.org/10.1002/1873-3468.13878 32620030

[33] Wik JA, Lundback P, la Cour Poulsen L, Haraldsen G, Skalhegg BS, Hol J. 3PO inhibits inflammatory NFkappaB and stress-activated kinase signaling in primary human endothelial cells independently of its target PFKFB3. PLoS One 2020, 15, e0229395. doi: https://doi.org/10.1371/journal.pone.0229395 32130250 PMC7055879

[34] Bedouhène S, Moulti-Mati F, Hurtado-Nedelec M, Dang PM-C, El-Benna J. Luminol-amplified chemiluminescence detects mainly superoxide anion produced by human neutrophils. Am J Blood Res 2017, 7, 41–8. 28804681 PMC5545213

[35] Metzler KD, Fuchs TA, Nauseef WM, Reumaux D, Roesler J, Schulze I, et al Myeloperoxidase is required for neutrophil extracellular trap formation: implications for innate immunity. Blood 2011, 117, 953–9. doi: https://doi.org/10.1182/blood-2010-06-290171 20974672 PMC3035083

[36] Björnsdottir H, Welin A, Michaëlsson E, Osla V, Berg S, Christenson K, et al Neutrophil NET formation is regulated from the inside by myeloperoxidase-processed reactive oxygen species. Free Radic Biol Med 2015, 89, 1024–35. doi: https://doi.org/10.1016/j.freeradbiomed.2015.10.398 26459032

[37] Kotowski K, Rosik J, Machaj F, Supplitt S, Wiczew D, Jablonska K, et al Role of PFKFB3 and PFKFB4 in cancer: genetic basis, impact on disease development/progression, and potential as therapeutic targets. Cancers (Basel) 2021, 13, 909. doi: https://doi.org/10.3390/cancers13040909 33671514 PMC7926708

[38] Weyand CM, Shen Y, Goronzy JJ. Redox-sensitive signaling in inflammatory T cells and in autoimmune disease. Free Radic Biol Med 2018, 125, 36–43. doi: https://doi.org/10.1016/j.freeradbiomed.2018.03.004 29524605 PMC6128787

[39] Weyand CM, Zeisbrich M, Goronzy JJ. Metabolic signatures of T-cells and macrophages in rheumatoid arthritis. Curr Opin Immunol 2017, 46, 112–20. doi: https://doi.org/10.1016/j.coi.2017.04.010 28538163 PMC5554742

[40] Liu D, Sun W, Zhang D, Yu Z, Qin W, Liu Y, et al Long noncoding RNA GSEC promotes neutrophil inflammatory activation by supporting PFKFB3-involved glycolytic metabolism in sepsis. Cell Death Dis 2021, 12, 1157. doi: https://doi.org/10.1038/s41419-021-04428-7 34907156 PMC8671582

[41] Baillet A, Hograindleur M-A, Benna JE, Grichine A, Berthier S, Morel F, et al Unexpected function of the phagocyte NADPH oxidase in supporting hyperglycolysis in stimulated neutrophils: key role of 6-phosphofructo-2-kinase. FASEB J 2016, 31, 663–73. doi: https://doi.org/10.1096/fj.201600720r 27799347

[42] Sakakibara R, Kato M, Okamura N, Nakagawa T, Komada Y, Tominaga N, et al Characterization of a human placental fructose-6-phosphate, 2-kinase/fructose-2,6-bisphosphatase. J Biochem 1997, 122, 122–8. doi: https://doi.org/10.1093/oxfordjournals.jbchem.a021719 9276680

[43] Xu J, Wang L, Yang Q, Ma Q, Zhou Y, Cai Y, et al Deficiency of myeloid pfkfb3 protects mice from lung edema and cardiac dysfunction in lps-induced endotoxemia. Front Cardio Med 2021, 8, 745810. doi: https://doi.org/10.3389/fcvm.2021.745810 PMC8511447 34660743

[44] Aquilano K, Baldelli S, Ciriolo MR. Glutathione: new roles in redox signaling for an old antioxidant. Front Pharmacol 2014, 5, 196. doi: https://doi.org/10.3389/fphar.2014.00196 25206336 PMC4144092

[45] Yoo HC, Yu YC, Sung Y, Han JM. Glutamine reliance in cell metabolism. Exp Mol Med 2020, 52, 1496–516. doi: https://doi.org/10.1038/s12276-020-00504-8 32943735 PMC8080614

[46] Pithon-Curi TC, Levada AC, Lopes LR, Doi SQ, Curi R. Glutamine plays a role in superoxide production and the expression of p47phox, p22phox and gp91phox in rat neutrophils. Clin Sci (Lond) 2002, 103, 403–8. doi: https://doi.org/10.1042/cs1030403 12241540

[47] Burmistrova O, Olias-Arjona A, Lapresa R, Jimenez-Blasco D, Eremeeva T, Shishov D, et al Targeting PFKFB3 alleviates cerebral ischemia-reperfusion injury in mice. Sci Rep 2019, 9, 11670. doi: https://doi.org/10.1038/s41598-019-48196-z 31406177 PMC6691133

[48] Emini Veseli B, Van Wielendaele P, Delibegovic M, Martinet W, De Meyer GRY. The PFKFB3 inhibitor AZ67 inhibits angiogenesis independently of glycolysis inhibition. Int J Mol Sci 2021, 22, 5970. doi: https://doi.org/10.3390/ijms22115970 34073144 PMC8198190

