# Supplementary material for: The small molecule inhibitor 3PO is a modulator of neutrophil metabolism, ROS production, and NET release
Source: Clin Exp Immunol. 2025 Feb 19;219(1):uxaf012. doi: 10.1093/cei/uxaf012 (PMC12060009; doi:10.1093/cei/uxaf012)
Supplement: uxaf012_suppl_Supplementary_Materials [file uxaf012_suppl_supplementary_materials.docx]

**The small molecule inhibitor 3PO is a modulator of neutrophil metabolism, ROS production and NET release**

Short title: 3PO modulates neutrophil metabolism, ROS and NET release

Michele Fresneda Alarcon^1^, Genna Ali Abdullah^1^, Andy Nolan^1^, Christina Linford^1^, Maria Martina Meschis^2^, Andrew L Cross^1^, Andrew Sellin^1^, Marie M Phelan^3,4^, Helen L Wright^1^

^1^Institute of Life Course and Medical Sciences, University of Liverpool, Liverpool, L7 8TX, UK

^2^Institute of Systems, Molecular and Integrative Biology (ISMIB), Liverpool Head and Neck Centre, University of Liverpool, Liverpool, L7 8TX, UK

^3^Institute of Systems Molecular and Integrative Biology, University of Liverpool, Liverpool, L69 7BE, UK

^4^High Field NMR Facility, Liverpool Shared Research Facilities University of Liverpool, Liverpool, L69 7TX, UK

Supplementary Figure 1. Effect of glucose availability on ROS and NET production by RA and HC neutrophils. Panels show raw (A) ROS luminescence and (B) NET fluorescence data (n = 4-5, * p<0.05, **p<0.01).
